# Ethanol and Acetaminophen Synergistically Induce Hepatic Aggregation and TCH346-Insensitive Nuclear Translocation of GAPDH

**DOI:** 10.1371/journal.pone.0160982

**Published:** 2016-08-11

**Authors:** Natasha T. Snider, Daniel A. Portney, Helen H. Willcockson, Dhiman Maitra, Hope C. Martin, Joel K. Greenson, M. Bishr Omary

**Affiliations:** 1 Department of Cell Biology and Physiology, University of North Carolina, Chapel Hill, NC, 27599, United States of America; 2 Department of Molecular & Integrative Physiology, University of Michigan, Ann Arbor, MI, 48109, United States of America; 3 Department of Pathology, University of Michigan, Ann Arbor, MI, 48109, United States of America; 4 Department of Internal Medicine, University of Michigan, Ann Arbor, MI, 48109, United States of America; 5 Veterans Administration Ann Arbor Health Care System, Ann Arbor, MI, 48105, United States of America; Medizinische Fakultat der RWTH Aachen, GERMANY

## Abstract

The glycolytic enzyme glyceraldehyde-3-phosphate dehydrogenase (GAPDH) signals during cellular stress via several post-translational modifications that change its folding properties, protein-protein interactions and sub-cellular localization. We examined GAPDH properties in acute mouse liver injury due to ethanol and/or acetaminophen (APAP) treatment. Synergistic robust and time-dependent nuclear accumulation and aggregation of GAPDH were observed only in combined, but not individual, ethanol/APAP treatments. The small molecule GAPDH-targeting compound TCH346 partially attenuated liver damage possibly via mitochondrial mechanisms, and independent of nuclear accumulation and aggregation of GAPDH. These findings provide a novel potential mechanism for hepatotoxicity caused by combined alcohol and acetaminophen exposure.

## Introduction

Chemically induced liver injury involves the formation of reactive intermediates, including electrophiles and oxygen free radicals, which can damage cellular structures and organelles and promote hepatocyte death [1, 2]. Alterations in protein post-translational modifications and formation of various types of oligomeric and misfolded protein species are common cellular responses to oxidative injury [3–5]. Some protein alterations carry functional consequences for cell fate and thus may provide opportunities to devise protective strategies against stress-induced cellular damage. To that end, the metabolic enzyme glyceraldehyde-3-phosphate dehydrogenase (GAPDH), which has homeostasis-related glycolytic roles as well as multiple stress- and toxicity-related functions [6–10], represents a potential target for pharmacological modulation in mitigating chemically induced liver injury [11].

GAPDH is a soluble cytoplasmic protein involved in carbohydrate metabolism by catalyzing the reversible oxidative phosphorylation of glyceraldehyde-3-phosphate. Another important function of GAPDH is in modulation of the cellular response to oxidative stress via its ability to aggregate in the cytoplasm or translocate to the nucleus—both activities are considered to be cell death-promoting [12–16]. Many of the molecular aspects, as well as co-regulators of the stress-related functions of GAPDH have been characterized in cell culture models and in a number of animal studies. For example, under conditions of oxidative stress and nitric oxide over-production, GAPDH becomes S-nitrosylated, binds to an E3 ubiquitin ligase (Siah1), and the complex translocates to the nucleus [17]. This process is thought to promote cell death by various transcriptional mechanisms, such as p53 activation [15]. Opposing cellular mechanisms to this pathway have also been reported, including antagonism of the GAPDH-Siah1 complex by B23/nucleophosmin and retention of GAPDH in the cytoplasm by RILP-like protein 1 (RILPL1), also known as GOSPEL [18, 19].

GAPDH is the only described molecular target for the antiapoptotic drug N-(benzo[b][1]benzoxepin-5-ylmethyl)-N-methylprop-2-yn-1-amine (also known as CGP 3466B, TCH346, and Omigapil). TCH346 (which is the name we use throughout this paper) is a structural analog of the monoamine oxidase B inhibitor R-(-)-deprenyl [20], with a molecular formula C_19_H_17_NO (PubChem ID: 6419718). Both TCH346 and deprenyl were found to block GAPDH S-nitrosylation, binding to Siah, and nuclear translocation [16]. Furthermore, TCH346 demonstrated protective properties in animal models of neurodegeneration [21–24]; with some exceptions such as in an mSOD1 model of amyotrophic lateral sclerosis where it did not provide a benefit [25].

We previously demonstrated that GAPDH undergoes nuclear translocation in isolated hepatocytes and *in vivo* during chronic mouse liver injury induced by the porphyrinogenic drug 3,5-diethoxycarbonyl-1,4-dihydrocollidine, which is associated with oxidative liver damage [11]. We also observed significant cytoplasmic and nuclear aggregation of GAPDH in liver explants from patients with alcoholic cirrhosis [11]. These findings led us to hypothesize that GAPDH functions as a sensor and an effector of liver injury, which we tested here in a model of acute liver injury due to acetaminophen overdose.

Acetaminophen (APAP) is a commonly used antipyretic and analgesic drug available as a single ingredient or in a formulation with other drugs. Although safe when used within the recommended doses, APAP is hepatotoxic in cases of overdose or when other risk factors, such as alcohol, are present [26]. APAP-related hepatotoxicity is due to its oxidative phase I metabolism by the cytochrome P450 enzymes (particularly CYP2E1) to the highly reactive intermediate N-acetyl-para-benzoquinoneimine (NAPQI). NAPQI generation leads to glutathione depletion and hepatocyte necrosis resulting from oxidative damage to mitochondria, nuclear DNA fragmentation and lipid peroxidation, among other factors [2].

Given the well-appreciated role of GAPDH in cellular damage due to oxidative stress, we sought to determine GAPDH involvement in oxidative liver damage due to APAP overdose in ethanol pre-treated mice. We also examined the effect of pharmacological targeting of GAPDH with TCH346.

## Materials and Methods

### Antibodies

The following antibodies were used: mouse anti-GAPDH [6C5], rabbit anti-lamin B1, rabbit anti-cytochrome c [EPR1327], and rabbit anti- carbamoyl phosphate synthetase-1 (CPS1) [EPR7493] (Abcam, Cambridge, UK); mouse anti-β-tubulin; mouse anti-pan actin, mouse anti-hsp60 clone LK2 and mouse anti-Bax (Thermo Scientific, Waltham, MA); and rabbit anti-SAPK/JNK (Cell Signaling, Danvers, MA).

### Liquid ethanol diet

We acclimatized 39 female C57BL/6J mice (12–14 weeks old) to a control liquid diet (Control Rodent Liquid Diet Lieber-DeCarli '82, Shake and Pour; Bio-Serv) for 3 days. On day 4 we divided the mice into a control group (n = 8), which continued to receive the control liquid diet for an additional 5 days, and an ethanol-fed group (n = 31) that received isocaloric ethanol-containing diet (Ethanol Maltose Dextrin Rodent Liquid Diet Lieber-DeCarli '82, Shake and Pour; Bio-Serv). In the latter group the ethanol concentration in the diet was increased from 1.67% on day 4, to 3.33% on day 5, to 5% on days 6–9. The ethanol-treated mice were further divided into 4 treatment groups, described below.

### Drug treatments, blood and tissue processing

The 31 ethanol pre-treated mice from the first experiment were divided into four new experimental groups according to treatment with APAP and TCH346, as follows: (i) ethanol control (n = 7), (ii) ethanol plus 500 mg/kg APAP treatment (n = 8), (iii) ethanol plus 500 mg/kg APAP + 0.2 mg/kg TCH346 (n = 8); and (iv) ethanol plus 500 mg/kg APAP + 1.0 mg/kg TCH346 (n = 8). APAP, alone or together with TCH346, was dissolved in 55°C sterile PBS, which was cooled to 37°C prior to injection. The mice were fasted for 8 hours (h) before intraperitoneal (IP) drug administration, and sacrificed by CO_2_ inhalation after 4h. Serum alanine aminotransferase (ALT) levels were measured at the Unit for Laboratory Animal Medicine core facility at the University of Michigan. The left liver lobe was apportioned into RNA-later, 10% buffered formalin, snap-frozen, or embedded in optimal cutting temperature (OCT) medium. All mice received humane care, and their use was approved by and performed in accordance with the University Committee on Use and Care of Animals (UCUCA) at the University of Michigan and the Institutional Animal Care and Use Committee (IACUC) at the University of North Carolina.

### Preparation of liver lysates, subcellular fractionation, and immunoblotting

Total liver lysates were prepared by homogenizing 25–50 mg of liver tissue in 1mL of 2X Tris-Glycine SDS sample buffer in the absence (non-reducing conditions; for detection of GAPDH aggregates) or presence (reducing conditions; for all other immunoblots) of 2-mercaptoethanol. Established protocols were followed for subcellular fractionation to obtain cytoplasmic and nuclear fractions [27] or mitochondria [28]. Mitochondrial Complex I activity was measured using a commercial kit (Cayman Chemical). The protein lysates were resolved on gradient 4%-20% SDS-PAGE (polyacrylamide gel electrophoresis) gels and transferred to polyvinylidene difluoride membranes, which were then blocked (in 5% milk in PBS/0.1% Tween-20) and incubated with the designated antibodies for immunoblotting.

### Immunofluorescence staining and confocal imaging

Fresh frozen OCT-embedded liver tissues were sectioned (6μM), fixed in -20°C acetone for 10 min, stained and imaged as previously described [11].

### Data analysis

Statistical analysis was done using Prism 6 (GraphPad Software). Photoshop (CS2; Adobe) was used for densitometry analysis of the immunoblots. Histological assessment of liver damage was performed by a trained pathologist (JKG) in a blinded fashion. All bar graphs represent means ± standard deviation.

## Results

### Ethanol/APAP co-treatment promotes nuclear accumulation of GAPDH in mouse liver

**Fig 1:**
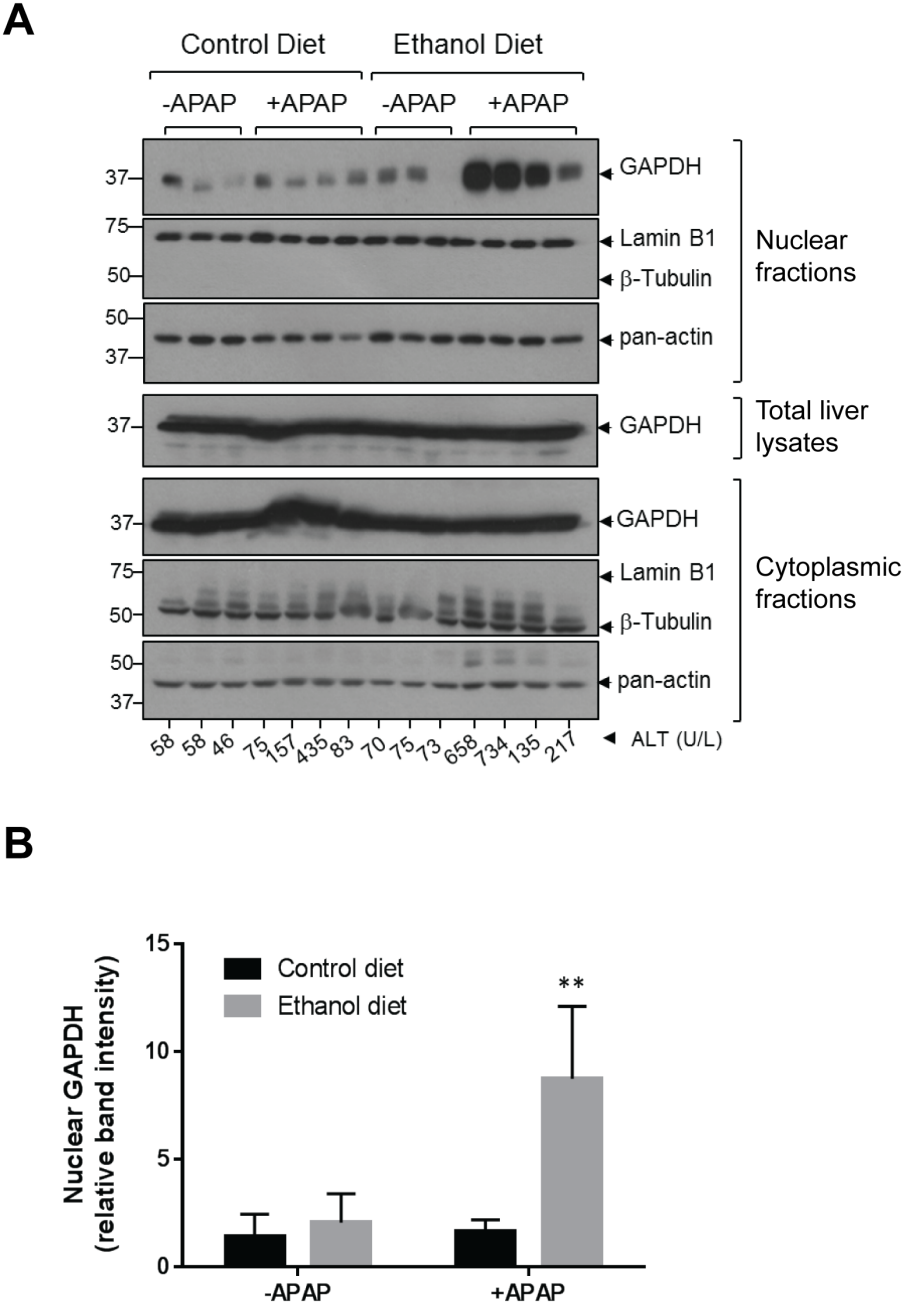
Ethanol and acetaminophen synergize to promote nuclear accumulation of GAPDH in the liver. **A**. Immunoblots of nuclear fractions (top 3 panels), total tissue lysates (middle panel) and cytoplasmic fractions (bottom 3 panels) from livers of female C57BL/6J mice. The mice were fed either a control or ethanol (EtOH)-containing liquid diet. The amount of EtOH (vol/vol) in the diet was: 0% on days 1–3; 1.67% on day 4; 3.33% on day 5; and 5% on days 6–9. The study was terminated on day 10, following an overnight fast of all mice and a single APAP injection (500mg/kg; during the last 4h), administered to the indicated groups. Note the EtOH/APAP-dependent redistribution of GAPDH in the nuclear compartment. A combined β-tubulin and lamin B1 blot was done to validate the separation of the cytoplasmic and nuclear fractions, while pan-actin served as loading control. **B**. Densitometric quantification of the relative levels of nuclear GAPDH from the three treatment groups shown in panel A. **, p<0.01, two-way ANOVA.

We tested the effect of short-term (6 day) ethanol pre-treatment on nuclear GAPDH accumulation in response to APAP (500mg/kg; 4h). While administration of either ethanol or APAP alone did not affect nuclear GAPDH levels, combination of the two treatments produced significant nuclear accumulation of GAPDH (Fig 1A). The ~8-fold increase of nuclear GAPDH (Fig 1B) in the combination, but not single treatments indicated synergistic effects of ethanol and APAP. The results were reproduced and quantified on an independent set of mice (8 mice/group), showing ~10 fold induction of nuclear GAPDH levels in EtOH/APAP-treated mice compared to control mice (not shown).

### APAP treatment following ethanol pre-exposure promotes aggregation of cytoplasmic GAPDH in a time-dependent manner

**Fig 2:**
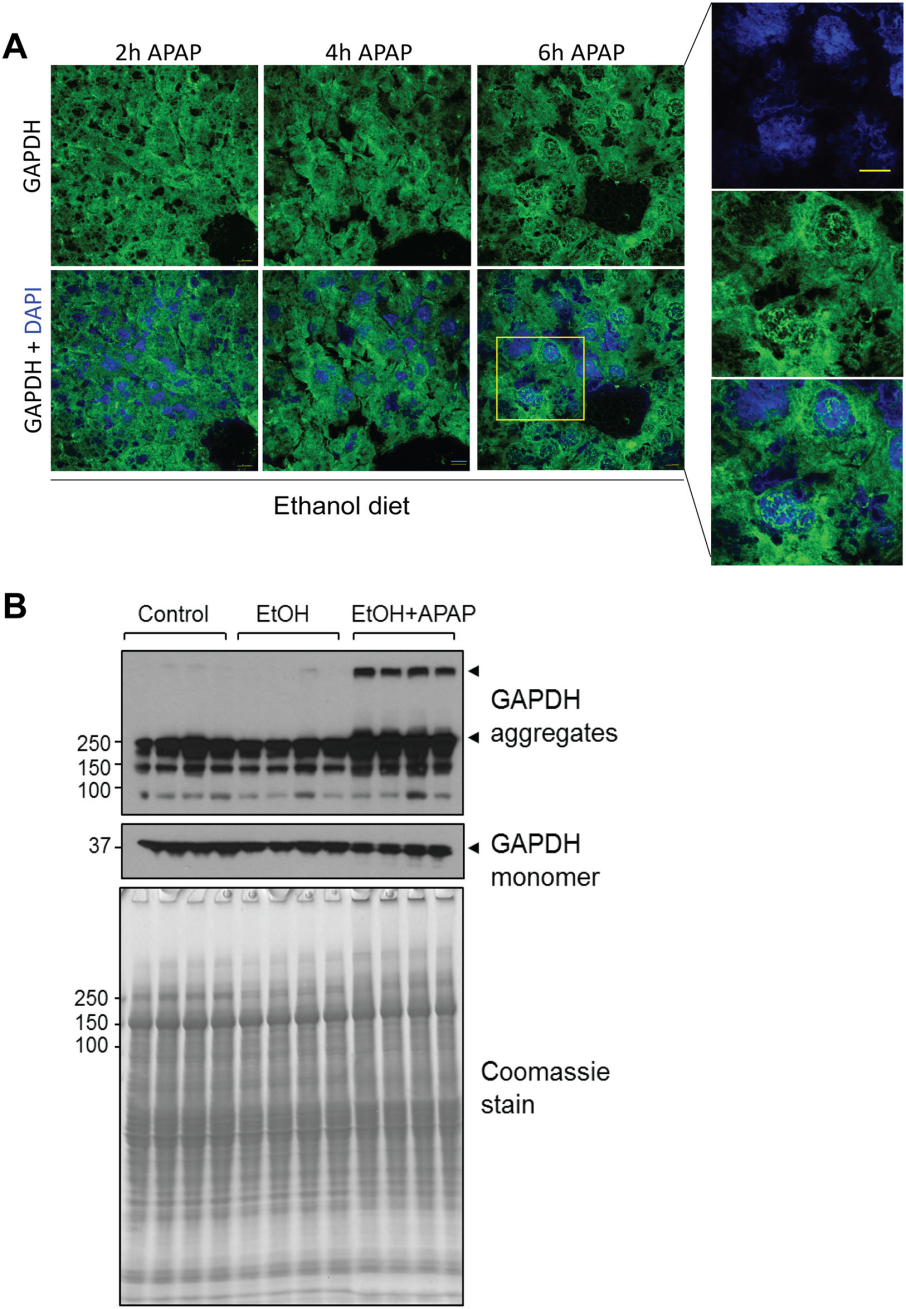
APAP promotes aggregation of cytoplasmic GAPDH in ethanol pre-treated mice. **A**. Indirect immunofluorescence analysis of GAPDH (green) together with DAPI-stained nuclei (blue) in livers from ethanol pre-treated mice that were given APAP for the indicated time periods. Inset highlights the presence of cytoplasmic and nuclear GAPDH aggregates 6h after APAP treatment (highlighted in the right 3 panels showing DAPI, GAPDH and combined staining). **B**. Total lysates of mouse livers from the indicated treatment groups were analyzed under non-reducing conditions by SDS-PAGE, followed by Coomassie stain or GAPDH immunoblot. The immunoblots showing GAPDH monomer and high molecular mass (HMM) complexes (arrowheads) were from the same membrane at different exposure times. Shown are 4 representative individual mice from each treatment group, out of 8 total mice/group.

Next we monitored the time-dependent effects of APAP administration on GAPDH in ethanol pre-treated mice. We noted significant nuclear presence of GAPDH at 4h and 6h post APAP injection (Fig 2A). Furthermore, upon APAP exposure GAPDH appeared aggregated in the cytoplasm and nuclei of ethanol-exposed mouse livers (Fig 2A). This was confirmed biochemically in the form of high molecular mass GAPDH complexes that migrated near the top of the SDS-PAGE gel (Fig 2B, arrowheads). The aggregates were only observed under non-reducing conditions (i.e., in the absence of 2-mercaptoehanol) indicating that they were produced via disulfide bond formation, a mechanism that was previously described to involve GAPDH active site cysteine residues [29].

### The GAPDH-targeting compound TCH346 partially attenuates combined ethanol/APAP liver injury relative to control livers

**Fig 3:**
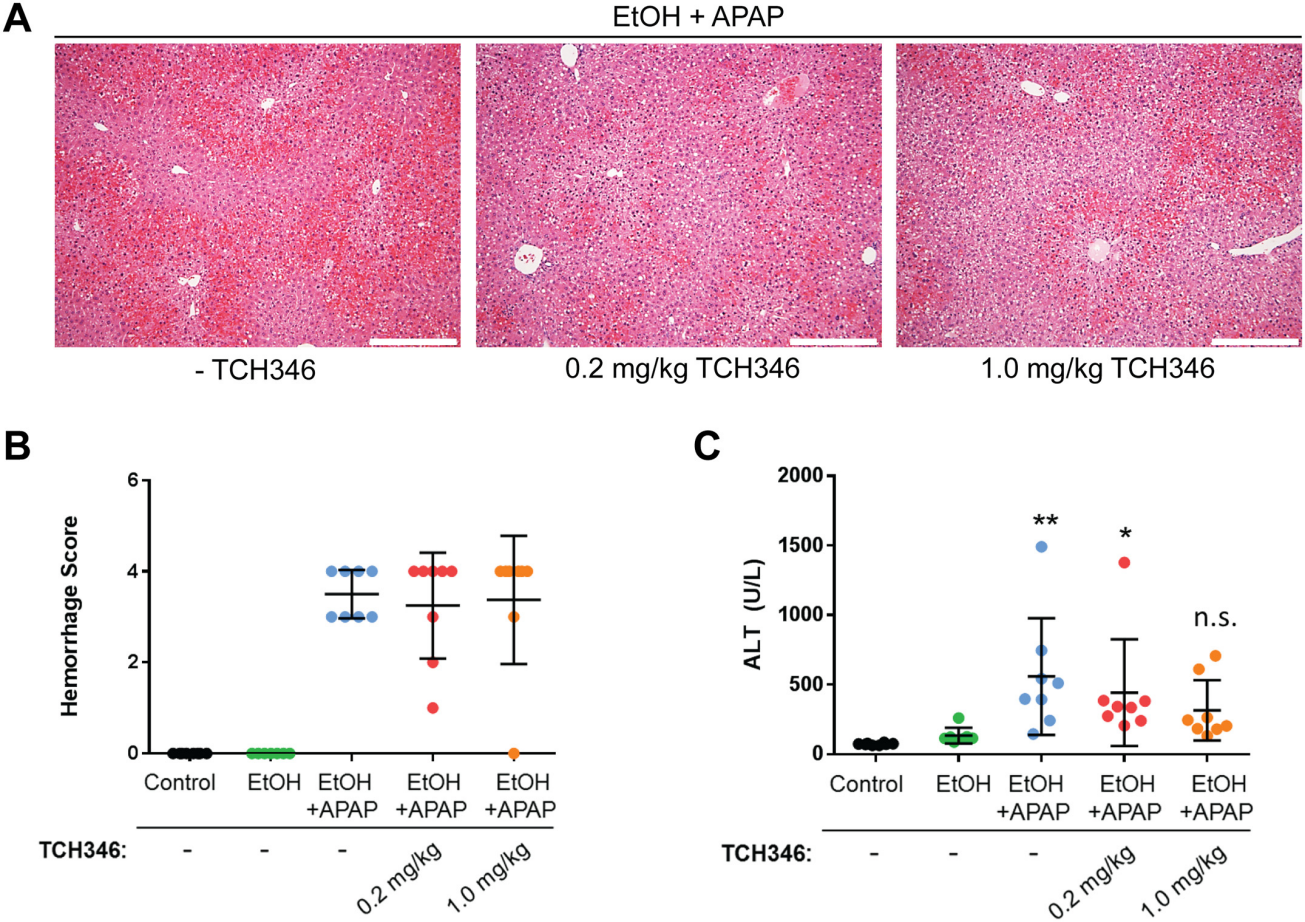
The GAPDH-targeting compound TCH346 partially attenuates combined ethanol and acetaminophen liver injury. **A**. Representative H&E staining images from livers of EtOH/APAP-treated mice in the absence (-TCH346) or presence of 0.2mg/kg or 1mg/kg TCH346. Scale bar = 200μm **B**. Histological scoring of the areas of hemorrhage in the different treatment groups. **C**. Serum ALT levels in mice receiving control liquid diet (Control) or EtOH-containing liquid diet in the absence/presence of 0.2 or 1 mg/kg TCH346, which was co-administered with APAP (500mg/kg for 4hr). *p<0.05; **p<0.01; n.s., not significant, relative to the control group (one-way ANOVA; Dunnet’s multiple comparisons test). There was no statistically significant difference in serum ALT between the EtOH/APAP groups receiving TCH346 as compared to EtOH/APAP alone.

To determine whether GAPDH functions as an effector of ethanol/APAP liver injury, we evaluated the effect of the small molecule TCH346. TCH346 was shown to be neuroprotective in animal models, in part by blocking the nuclear GAPDH-mediated cell death cascade [16]. We compared the effect of a low (0.2 mg/kg) and higher (1 mg/kg) dose of TCH346. Doses were selected based on demonstrated effectiveness of the compound in previous *in vivo* studies in neuronal injury models [16]. Histological analysis of the livers revealed the presence of combined EtOH/APAP-induced hemorrhage in all treatment groups (Fig 3A and 3B), while serologic ALT (Fig 3C) but not histologic prominent evidence of necrosis was observed. However, serum analysis revealed partial and dose-dependent TCH346-mediated hepatoprotection, as evidenced by reduced ALT levels, when compared with the control group (Fig 3C). Since the protection was only noted when comparing ALT but not hemorrhage changes with controls, the biologic significance of the protection is unclear.

### TCH346 treatment does not prevent nuclear translocation and aggregation of GAPDH in the combined ethanol/APAP injury model

**Fig 4:**
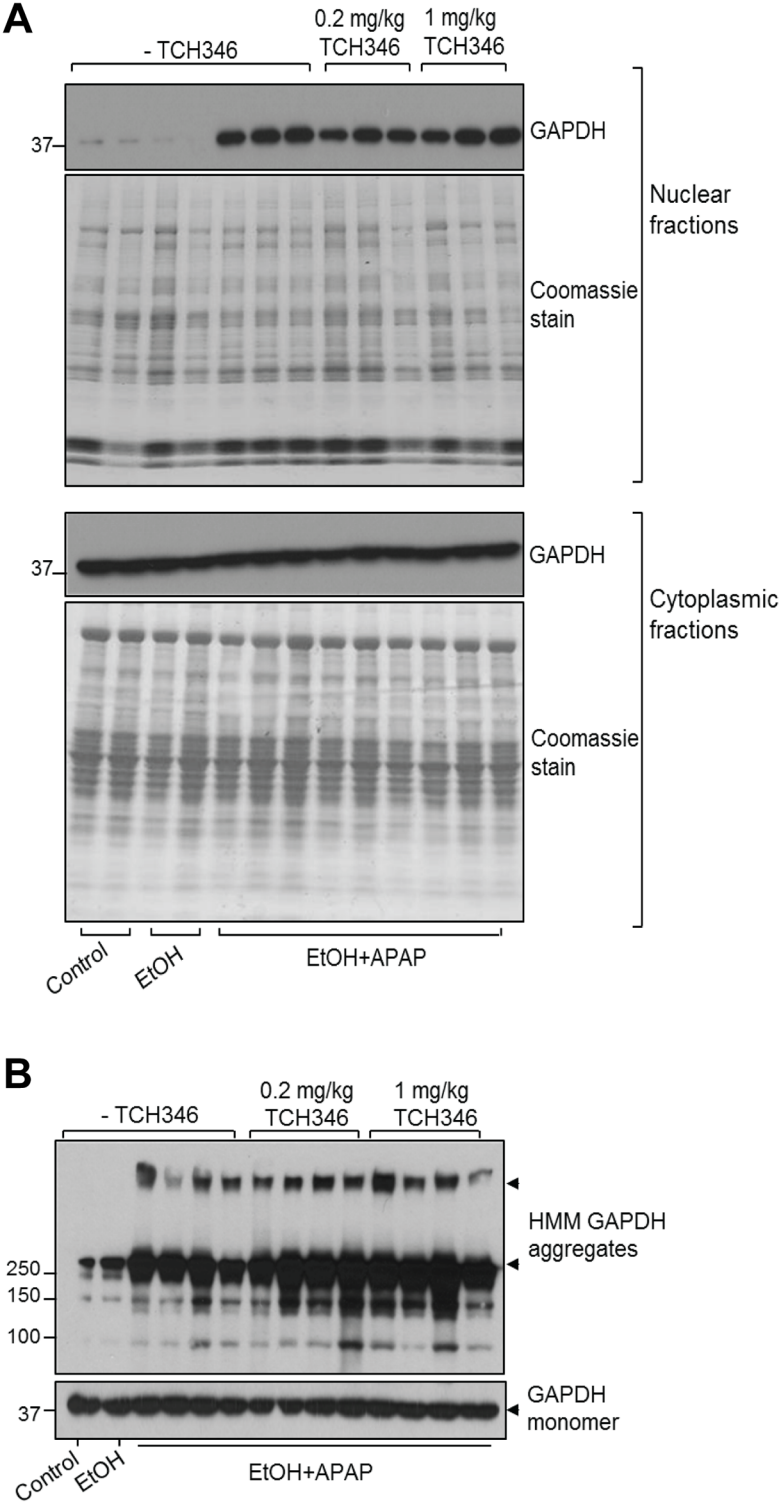
TCH346 treatment does not alter the nuclear levels or aggregation of GAPDH in the livers from mice treated with ethanol/APAP. **A**. Coomassie stain and GAPDH immunoblot of nuclear and cytoplasmic fractions from representative livers of control (n = 2), EtOH (n = 2) and EtOH/APAP (n = 9)-treated mice, as labeled at the bottom. The mice in the latter group (last nine lanes) represent those receiving no additional treatment (-TCH346), 0.2mg/kg TCH346 or 1mg/kg TCH346, as labeled at the top. Shown are 2 or 3 representative individual mice from each treatment group, out of 8 total mice/group. **B**. GAPDH immunoblot (non-reducing conditions) of total lysates from livers of 1 each of control and EtOH-treated mice, and 12 EtOH+APAP- treated mice, as labeled at the bottom. The mice in the latter group (last 12 lanes) represent those receiving no additional treatment (-TCH346), 0.2mg/kg TCH346 or 1mg/kg TCH346, as labeled at the top. Shown are 1 or 4 representative individual mice from each treatment group, out of 8 total mice/group.

The previously reported protective effects of TCH346 treatment were linked to its ability to prevent the nuclear translocation of GAPDH [16], which otherwise leads to the induction of several pro-apoptotic genes [15]. Therefore, we tested whether the decrease in serum ALT levels in the TCH346-treated mice, particularly at the higher dose, correlated with a decrease in GAPDH aggregation and nuclear presence. To our surprise, neither parameter was altered by TCH346, regardless of the dose administered (Fig 4). These data suggest that, unlike in brain, the partial protective effects of TCH346 in the liver are independent of its activity on nuclear GAPDH.

### TCH346 treatment preserves mitochondrial Complex 1 activity and cytochrome c levels

**Fig 5:**
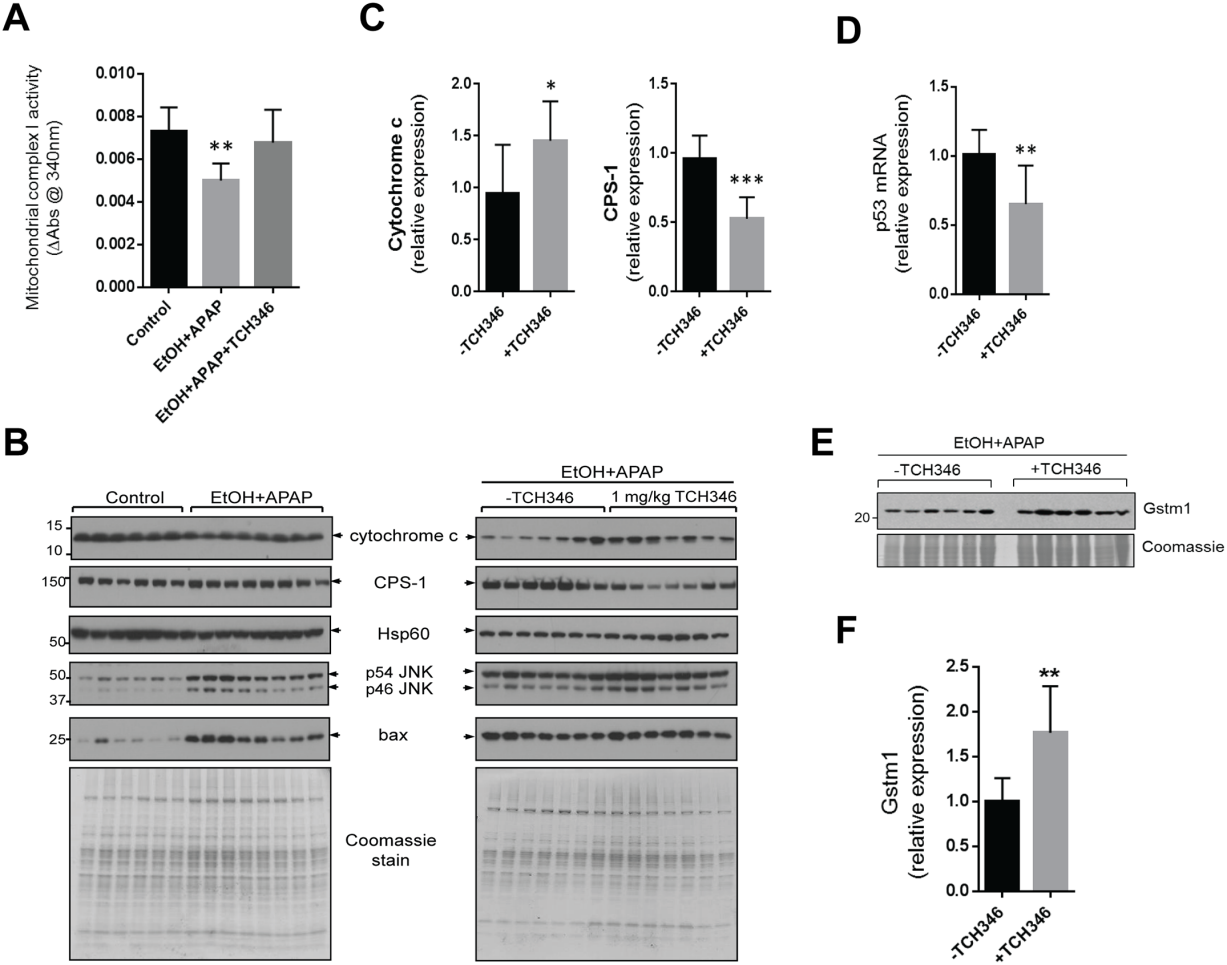
TCH346 treatment preserves mitochondrial Complex I activity and cytochrome c levels, and decreases mitochondrial CPS-1 in livers from EtOH+APAP-treated mice. **A**. Measurement of Complex I activity (rate of NADH oxidation measured as a decrease in absorbance at 340nm) in liver mitochondrial extracts of the designated treatment groups. **p<0.01 compared to control group (one-way ANOVA; Dunnet’s multiple comparisons test). **B**. Coomassie gel stains and immunoblots for the indicated proteins in the mitochondrial fractions of control vs. EtOH/APAP (left panels), or—TCH346 versus 1mg/kg TCH346 in EtOH/APAP-treated mice (right panels). TCH346 did not affect the EtOH+APAP-induced increased presence of JNK or Bax, but was associated with increased levels of cytochrome c and decreased levels of the mitochondrial matrix protein CPS-1. **C**. Densitometric quantification of cytochrome c and CPS-1 levels from panel B (right; EtOH+APAP with or without TCH346). *p<0.05; ***p<0.001 when comparing +TCH346 to the -TCH346 group (unpaired t-test). **D**. Downregulation of p53 mRNA by TCH346. **E**. Upregulation of the antioxidant protein Gstm1 in livers from TCH346-treated mice. **F**. Quantification of the immunoblot in panel E. **p<0.01 compared to the -TCH346 group; unpaired t-test. TCH46 dose was 1mg/kg in all experiments shown.

APAP is known to promote hepatocyte necrosis via mitochondrial damage mediated by protein translocation into and out of the mitochondria [30, 31]. To test whether TCH346 affected mitochondrial function, we examined activity of mitochondrial Complex I. APAP administration to ethanol pre-treated mice caused ~25% decrease in Complex I activity, which was reversed to control levels by TCH346 treatment (Fig 5A). Immunoblot analysis of isolated liver mitochondria demonstrated that ethanol/APAP-treated mice that also received TCH346 had higher levels of cytochrome c and lower levels of the highly abundant mitochondrial matrix protein carbamoyl phosphate synthase 1 (CPS-1) (Fig 5B and 5C). Since mitochondrial cytochrome c levels are sensitive to oxidative stress mediated via p53 [32] and glutathione depletion [33], we assessed the effect of TCH346 on p53 mRNA and glutathione-S-transferase M1 (Gstm1) protein. TCH346 reduces the levels of p53 mRNA (Fig 5D) and increases Gstm1 expression (Fig 5E and 5F), suggesting that the increased levels of mitochondrial cytochrome c in response to TCH346 may be a result of increased resistance to oxidative stress-induced damage. Another prominent target of mitochondrial oxidative stress is CPS-1, a component of the urea cycle and a potential serum biomarker of acute mouse and human liver injury [34], which is reduced by TCH346. These data demonstrate that TCH346 alters mitochondrial protein dynamics during ethanol/APAP-induced liver injury, but the exact mechanisms remain to be determined.

## Discussion

Formation of reactive metabolites that are damaging to cellular organelles is a common mechanism in drug-induced liver injury, including acetaminophen overdose. Understanding the downstream mechanisms of chemically induced hepatotoxicity can reveal potential treatment modalities that may prevent or curtail acute liver failure. The present study provides evidence that the glycolytic enzyme GAPDH is a prominent cellular target in mouse liver injury due to combined ethanol and APAP exposure. We demonstrate that GAPDH undergoes significant nuclear translocation and aggregation into high molecular weight complexes in response to combined ethanol and APAP treatment. Using a pharmacological approach aimed at targeting the nuclear translocation of GAPDH, we demonstrated a modest protective effect of the drug TCH346. However, the hepatoprotective mechanism of TCH346 was independent of nuclear GAPDH translocation and aggregation, in contrast to previous studies on this compound in models of neuroprotection [16, 20]. However the TCH346 effects in the liver were likely mediated in part via mitochondrial mechanisms. Specifically, TCH346 treatment preserved Complex I activity and increased levels of mitochondrial cytochrome c. The latter is known to act as a mediator of APAP-related hepatotoxicity upon its stress-induced release from mitochondria [35, 36].

CPS-1 catalyzes the first step of the urea cycle by synthesizing carbamoyl phosphate from ammonia and bicarbonate. Since carbamoyl phosphate is converted to citrulline, CPS-1 also affects the availability of precursors for NO synthesis [37], which may indirectly affect GAPDH S-nitrosylation. Furthermore, a recent study identified a large number of mitochondrial proteins that undergo pathology-promoting nitration in response to APAP mouse liver injury and among these, CPS1 (which contains 36 tyrosines in the mouse isoform) had the greatest number of nitrotyrosine-containing peptides [144] that were detected by mass spectrometry [38]. Although the effect of nitration on CPS1 specifically was not investigated, nitration is known to generally inhibit the activities of metabolic enzymes, leading to mitochondrial dysfunction [38, 39]. Therefore another possibility is that TCH346 may protect from mitochondrial damage by promoting the release of the highly abundant enzyme CPS-1 that may have been rendered dysfunctional by stress-induced nitration.

GAPDH is one of many protein targets for stress-induced S-nitrosylation, which is the coupling of an NO moiety to a reactive cysteine thiol to form S-nitrosothiol. The function of S-nitrosylation is dependent on the stimulus as well as the protein target. In the case of GAPDH, this post-translational modification promotes GAPDH binding to the E3 ubiquitin ligase Siah 1, which contains a nuclear localization signal and acts as a vehicle to promote the nuclear accumulation of a small pool of GAPDH [17]. Among the known functions of nuclear GAPDH are activation of p300/CREB-binding protein and its target, p53, to induce apoptotic cell death [15, 40] as well as trans-nitrosylation of nuclear resident proteins [41]. Given that apoptosis is not thought to be a major contributor to APAP-induced hepatotoxicity [42], the transcriptional pathway of nuclear GAPDH signaling, as related to apoptosis, is unlikely to be involved. One possibility is that GAPDH-mediated trans-nitrosylation of nuclear targets, including the deacetylase sirtuin 1 [41, 43] could mediate APAP liver injury, based on the known importance of another sirtuin family member, the mitochondrial sirtuin 3, in APAP liver injury [44].

TCH346 was shown to target GAPDH [20] and to prevent its Siah1-mediated translocation [16]. Studies in animal models of neurodegeneration demonstrated TCH346-mediated protection [22, 23, 45], although this protection did not translate to clinical trials of Parkinson Disease and ALS [46, 47]. Nevertheless, given its favorable safety profile that was shown in the clinical studies, there is potential to repurpose TCH346 (also known as CGP 3466 and Omigapil) in other disease settings. To that end, TCH346 is currently being considered as a potential treatment in congenital muscular dystrophies in a clinical trial (NCT01805024), based on its effects in preclinical models [48–50]. Our study adds hepatoprotection to the repertoire of other potential uses for TCH346, although its utility in this setting is not as profound as noted in the brain and will need to be validated in additional models of drug-induced liver injury. Dosage and timing of administration are two important considerations for future investigation. This is illustrated by previous studies showing that the *in vivo* neuroprotective effects of TCH346 are highly dose-dependent and exhibit a bell-shaped curve, with the protective effect being greatest at 0.1mg/kg and lost at or above 1mg/kg (45). The contribution of hepatic metabolism to TCH346 activity in the liver will also need to be examined in more detail. Our findings that TCH346 exerts its partial protective effects by targeting mitochondria are consistent with some of the previously reported neuroprotective properties of TCH346. For example, in the *pmn/pmn* (progressive motor neuropathy) mouse model of ALS, administration of TCH346 extended life-span, prevented weight loss, and slowed the loss of motor neurons by preserving mitochondrial integrity, although the molecular events by which TCH346 protected mitochondria were not delineated [23].

**Fig 6:**
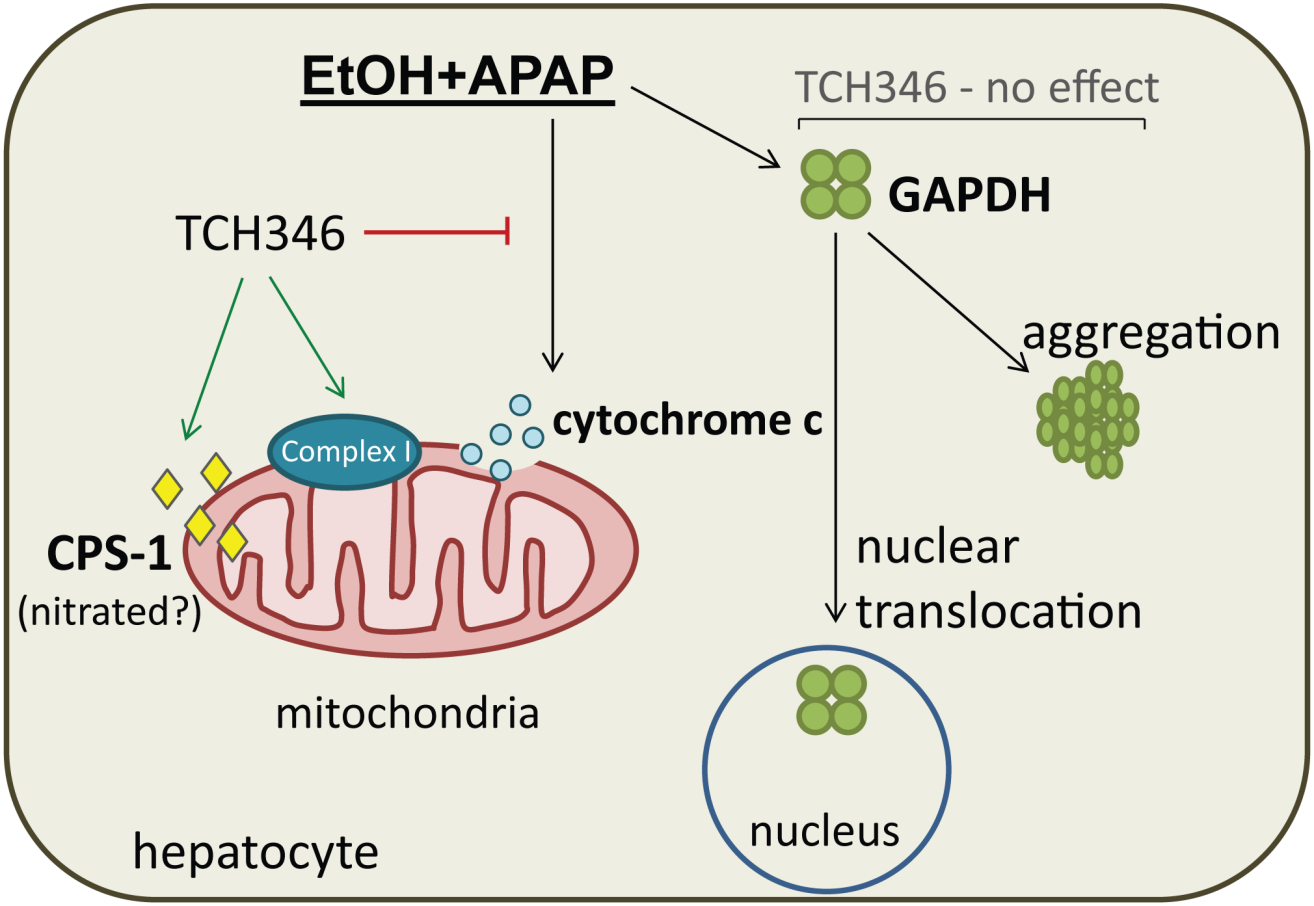
Summary of liver GAPDH changes in response to combined ethanol and APAP exposure and the effects of TCH346 treatment. Combined ethanol and APAP mediated mouse liver injury is associated with a significant increase in GAPDH nuclear translocation and cytoplasmic aggregation. The drug TCH346, which was previously shown to target GAPDH in the brain, attenuates liver injury in association with EtOH+APAP treatment. The mechanism by which TCH346 exerts its effect in this liver injury model are independent of GAPDH nuclear levels and aggregation, but involve mitochondrial events. TCH346 treatment is associated with increased mitochondrial Complex I activity and cytochrome c levels, and decreased CPS-1, which may be nitrated and/or non-functional [38].

In summary, we report on a novel signaling mechanism involved in ethanol/APAP liver injury that converges on the nuclear translocation and aggregation of GAPDH. The small molecule GAPDH-targeting compound TCH346 did not reverse nuclear accumulation of GAPDH in response to ethanol/APAP treatment, but showed modest hepatoprotection that was potentially mediated by mitochondrial mechanisms (Fig 6).

## Abbreviations

ALT: alanine aminotransferase
APAP: acetaminophen
CPS1: carbamoyl phosphate synthetase-1
EtOH: ethanol
GAPDH: glyceraldehyde-3-phosphate dehydrogenase
NAPQ1: N-acetyl-para-benzoquinoneimine
NO: nitric oxide
OCT: optimal cutting temperature
TCH346: *N*-(Dibenz[*b*,*f*]oxepin-10-ylmethyl)-*N*-Methyl-*N*-(2-propynyl)amine maleate (GAPDH-targeting small molecule drug)
